# Effects of Virtual Reality–Based Physical Exercise Interventions on Behavioral, Executive Function, and Motor Outcomes in Children and Adolescents With Autism Spectrum Disorder: Systematic Review and Meta-Analysis

**DOI:** 10.2196/98579

**Published:** 2026-07-23

**Authors:** Yaxiang Jia, Kai Qi, Kelong Cai, Ye Mao, Hui Guo, Qiyi Wang, Aiguo Chen

**Affiliations:** 1 College of Physical Education, Yangzhou University, Yangzhou City, No. 88 Daxue South Road, Yangzhou, Jiangsu, China; 2 School of Sport and Brain Health, Nanjing Sport Institute, No. 8, Linggusi Road, Xuanwu District, Nanjing, Jiangsu, China, 86 025-84755150; 3 Gdansk University of Physical Education and Sport, Poland, Pomerania, Poland; 4 Department of Endocrinology, Drum Tower Hospital Affiliated to Nanjing University Medical School, Nanjing, Jiangsu, China; 5 School of Psychology, Beijing Sport University, Beijing, Beijing, China; 6 School of Psychology, Shanghai University of Sport, Shanghai, Shanghai, China

**Keywords:** autism spectrum disorder, physical exercise, virtual reality, executive function, motor performance, behavior

## Abstract

**Background:**

In addition to core behavioral symptoms, children and adolescents with autism spectrum disorder (ASD) frequently exhibit impairments in executive function and motor performance. Although virtual reality (VR)–based physical exercise interventions are increasingly used in ASD rehabilitation, evidence regarding their multidimensional effects remains limited.

**Objective:**

This study aimed to systematically review the effects of VR-based physical exercise interventions on behavioral outcomes, executive function, and motor performance in children and adolescents with ASD.

**Methods:**

PRISMA (Preferred Reporting Items for Systematic Reviews and Meta-Analyses) 2020 guidelines were followed. PubMed (National Library of Medicine), Embase (Elsevier), Web of Science, Scopus (Elsevier), and other databases were searched from inception to May 7, 2026. Eligible studies included randomized and nonrandomized trials involving participants aged 6‐18 years with ASD. The interventions consisted of VR-based exercise programs involving physical activity participation, including active video games, motion-sensing interactive training, and augmented reality–based exercise training. Risk of bias was assessed using Risk of Bias 2 (RoB 2; Cochrane Bias Methods Group) and Risk of Bias in Nonrandomized Studies of Interventions (ROBINS-I; Cochrane Bias Methods Group), and certainty of evidence was evaluated using the Grading of Recommendations, Assessment, Development, and Evaluation (GRADE) framework. Random-effects meta-analyses were conducted using Hedges *g* standardized mean differences (SMDs).

**Results:**

A total of 15 studies involving 439 children and adolescents with ASD were included; among them, 9 studies were eligible for meta-analysis. Behavioral outcomes were reported in only 3 studies. Owing to substantial heterogeneity in study designs and assessment instruments, we did not conduct a meta-analysis for these outcomes. Current evidence suggests inconsistent effects on social interaction and stereotyped behaviors. VR-based physical exercise interventions may improve executive function (SMD 0.75, 95% CI 0.32‐1.18; *I*²=0.0%; *P*=.01; GRADE moderate). However, the prediction interval crossed the line of no effect (−0.12 to 1.63). VR-based physical exercise interventions were associated with improvements in motor performance (SMD 1.08, 95% CI 0.08‐2.08; *I*²=74.3%; *P*=.04; GRADE low). However, the wide prediction interval suggests substantial between-study variability (−1.31 to 3.47).

**Conclusions:**

Current evidence suggests a relatively consistent positive effect of VR-based physical exercise interventions involving physical activity participation on executive function in children and adolescents with ASD. However, substantial uncertainty remains regarding their effects on motor performance and behavioral outcomes. Unlike previous reviews that primarily focused on general VR interventions, social skills training, or single functional outcomes, this systematic review specifically examined the multidimensional effects of VR-based physical exercise interventions. The findings suggest that VR-based physical exercise interventions may be implemented as adjuncts to conventional exercise or rehabilitation programs rather than as stand-alone interventions. Future large-scale, high-quality randomized controlled trials with larger sample sizes, standardized intervention reporting, and long-term follow-up are needed to further clarify the optimal implementation conditions and underlying mechanisms of different forms of VR-based exercise training.

## Introduction

Autism spectrum disorder (ASD) is a lifelong neurodevelopmental condition characterized by persistent impairments in social communication and social interaction, alongside restricted and repetitive patterns of behavior, interests, or activities [1]. These core features typically emerge in early childhood and persist across the lifespan, substantially affecting learning, social participation, and quality of life. Beyond these core features, growing evidence indicates that children and adolescents with ASD frequently exhibit impairments across multiple functional domains, including executive function and motor abilities [2]. These impairments are closely associated with core behavioral symptoms and may contribute to broader functional limitations. Executive function refers to a set of higher-order cognitive processes that support complex, goal-directed behaviors. It encompasses 3 core components—working memory, inhibitory control, and cognitive flexibility—that collectively regulate behavior and modulate sensory processing and performance [3]. Deficits in executive function may lead to cognitive and emotional dysregulation and may exacerbate difficulties in social interaction and repetitive or stereotyped behaviors by undermining behavioral control [4,5]. Concurrently, motor impairments in children and adolescents with ASD manifest as deficits in postural control [6], balance [7], and motor coordination [8], as well as delayed or atypical development of gross and fine motor skills. Evidence suggests that limited motor competence not only restricts opportunities for engagement in physical exercise and physical activity but may also exert long-term adverse effects on behavioral outcomes and psychological development by constraining social participation and adaptive functioning [9,10]. Therefore, impairments in core behaviors, executive function, and motor performance are not independent but dynamically interact to influence overall functional performance and developmental trajectories in children and adolescents with ASD.

Among existing intervention strategies, pharmacological and behavioral interventions play important roles in alleviating core symptoms of ASD. However, these approaches often require substantial professional support and long-term investment and remain limited by poor adherence and uncertain long-term effectiveness [11]. In contrast, physical exercise interventions have gained increasing attention as nonpharmacological approaches due to their favorable safety profiles, relatively low cost, and feasibility in naturalistic settings. Previous studies indicate that diverse forms of physical exercise, including basketball, swimming, Tai Chi, and equine-assisted activities, may positively influence social interaction skills, behavioral outcomes, and motor function in children and adolescents with ASD [12-16]. By integrating physical activity with contextual interaction, physical exercise may facilitate physical functioning while providing structured opportunities for social interaction and behavioral regulation, thereby supporting their use as part of comprehensive interventions for children and adolescents with ASD [17].

Although conventional physical exercise interventions have demonstrated potential benefits, their long-term implementation in children and adolescents with ASD remains challenging because of poor adherence, limited motivation, heightened sensitivity to environmental stimuli, and difficulties tailoring interventions to individual ability levels [1,2,18]. These limitations may compromise the sustainability of intervention effects and limit the feasibility of large-scale implementation. Consequently, researchers have increasingly explored more structured, controllable, and engaging exercise interventions to improve participation experiences and training outcomes in children and adolescents with ASD.

Against this background, virtual reality (VR)–based physical exercise interventions have emerged as a promising complement to conventional exercise interventions [16,19]. By integrating multisensory stimuli, including visual, auditory, and motion-based interactions, VR technology can provide immersive, interactive, and controllable training environments for children and adolescents with ASD. These environments may reduce interference from complex social stimuli in real-world settings while increasing engagement during training [20-22]. The VR-based physical exercise interventions examined in this systematic review primarily included VR-based or motion-interactive training modalities involving active participation in physical activity, such as active video games (AVGs) [23], exergames [24], and virtual sports activities [25]. These interventions may facilitate motor learning and motor skill development through real-time feedback and adaptive difficulty adjustment. They may also reduce anxiety and avoidance behaviors through structured and predictable training environments, thereby supporting behavioral regulation and social participation [26]. Several systematic reviews have evaluated the effectiveness of VR interventions in individuals with ASD. For example, Mittal et al [27] examined the effects of immersive VR technologies on cognitive, social, and emotional functioning in children and adolescents with ASD. However, the included interventions were not restricted to exercise-based approaches, and few eligible studies were included. In addition, Yang et al [28] conducted a systematic review on the effects of VR interventions on social skills in children and adolescents with ASD. However, the included interventions were not centered on physical exercise, and no meta-analysis was conducted. Although the review by Hocking et al [23] included exercise-related VR interventions, it primarily focused on the effects of AVGs on motor function in individuals with developmental disabilities. It did not specifically focus on children and adolescents with ASD or evaluate multidimensional outcomes comprehensively, such as behavioral outcomes and executive function.

In recent years, increasing evidence has suggested that VR-based physical exercise interventions may improve balance, gross motor skills, and certain aspects of executive function in children and adolescents with ASD [5,7,29]. Nevertheless, substantial heterogeneity remains across studies regarding VR device types, exercise modalities, intervention duration, and outcome measures. As a result, integrated evidence on the multidimensional effects of VR-based physical exercise interventions in children and adolescents with ASD remains limited. Although the number of available studies remains limited, a comprehensive evaluation of this intervention approach is warranted to determine whether VR-based physical exercise interventions can serve as alternative or adjunctive exercise interventions for children and adolescents with ASD.

Therefore, this systematic review aimed to systematically review and meta-analyze the available evidence on the effects of VR-based physical exercise interventions on behavioral outcomes, executive function, and motor performance in children and adolescents with ASD. Unlike previous reviews, this systematic review specifically focused on VR interventions centered on active participation in physical activity and integrated outcome measures across multiple functional domains to clarify the potential clinical value of these interventions in children and adolescents with ASD. Clarifying the effectiveness of VR-supported exercise interventions may help inform the development of accessible and scalable digital rehabilitation strategies for children and adolescents with ASD.

## Methods

### Protocol and Registration

The protocol for this systematic review and meta-analysis was preregistered in the PROSPERO (International Prospective Register of Systematic Reviews; CRD420251266730). No deviations from the registered protocol were identified during the review process. This review was conducted and reported in accordance with the PRISMA (Preferred Reporting Items for Systematic Reviews and Meta-Analyses) 2020 statement (Checklists 1 and 2) [30].

### Eligibility Criteria

The eligibility criteria were established in accordance with the Population, Intervention, Comparison, Outcomes, and Study design (PICOS) framework.

- Population: children and adolescents aged 6‐18 years with a clinical diagnosis of ASD.
- Intervention: studies using VR-based physical exercise as the primary intervention were eligible for inclusion. VR-based physical exercise interventions were defined as structured and planned physical activities delivered via immersive or nonimmersive VR systems, including, but not limited to, virtual sports games and motion-based video games, in which physical activity constituted the core component of the intervention. Interventions meeting this definition were included regardless of VR immersion level, exercise modality, supervision, intervention setting, intensity, frequency, or duration.
- Comparison: eligible studies compared VR-based physical exercise interventions with usual care and no intervention.
- Outcomes: studies reporting at least one relevant outcome were included. Outcomes encompassed core behavioral measures (eg, repetitive or stereotyped behaviors, social communication and interaction abilities), executive function, and motor-related outcomes (eg, motor skills, balance, or coordination), assessed using validated or widely accepted instruments.
- Study design: both randomized controlled trials (RCTs) and non-RCTs were included.

The exclusion criteria were as follows: (1) nonoriginal studies, including reviews, commentaries, conference abstracts, and case reports; (2) studies in which participants were not exclusively children or adolescents diagnosed with ASD; (3) interventions that did not involve VR-based exercise or physical activity components; (4) studies that did not report prespecified behavioral, executive function, or motor performance outcomes; and (5) studies that did not meet the predefined eligibility criteria for study design, such as single-case studies. These exclusion criteria were established to ensure sufficient specificity and comparability across studies regarding participant characteristics, intervention modalities, and outcome measures, thereby supporting subsequent evidence synthesis and meta-analysis.

Studies with extractable quantitative data were synthesized using meta-analysis, whereas studies lacking sufficient data for effect size calculation were summarized descriptively using qualitative synthesis methods. When relevant outcomes were reported but data were incomplete or could not be directly extracted, corresponding authors were contacted, when feasible, to obtain missing data. Studies for which complete data remained unavailable were documented accordingly and included only in the qualitative synthesis.

### Information Sources

To comprehensively identify relevant studies, systematic searches were conducted in the following electronic databases and search platforms: PubMed (National Library of Medicine), Embase (Elsevier), PsycINFO (EBSCOhost), Scopus (Elsevier), Web of Science Core Collection (Clarivate), ProQuest, ScienceDirect, and IEEE Xplore. The final search was conducted on May 7, 2026. In addition to electronic database searches, gray literature sources and clinical trial registries, including ProQuest Dissertations and Theses and ClinicalTrials.gov, were searched to reduce the risk of publication bias. Backward citation searching of the reference lists of included studies and relevant review articles was also performed, alongside forward citation searching through Web of Science. Manual searches were also conducted to identify additional relevant studies.

### Search Strategy

The search strategy was developed using a combination of controlled vocabulary terms (eg, MeSH and Emtree terms) and free-text keywords and was adapted to the search syntax and requirements of each database. The search strategy incorporated the following key concepts: (1) ASD; (2) VR; (3) physical exercise or physical activity; and (4) behavioral, executive function, and motor outcomes. To maximize search sensitivity and specificity, Boolean operators (AND/OR) were used to combine search concepts, and database-specific subject headings were applied, when appropriate, to broaden the search scope. The complete and reproducible search strategies, including all search terms, limits, and filters, are provided in (S1 in Multimedia Appendix 1).

Only English-language full-text articles were included. No automated search translation tools, natural language processing techniques, or text-mining tools were used to generate or optimize search terms. All search strategies were manually adapted to the characteristics of each database. All retrieved records were imported into EndNote (Clarivate Plc) for reference management and duplicate removal before study screening. Before implementation, the search strategies underwent internal review to ensure the completeness and accuracy of the search syntax and content.

### Selection Process

Two reviewers (YJ and KQ) independently conducted study selection using a 2-stage screening procedure. First, titles and abstracts were screened to identify potentially eligible studies, followed by full-text review to determine final eligibility. All retrieved records were imported into EndNote for reference management and duplicate removal. During screening, both reviewers independently completed each stage and documented the reasons for full-text exclusion. Any disagreements regarding study eligibility were resolved through discussion with a third reviewer (KC) until consensus was reached. Because only English-language full-text studies were included, no translation procedures were required. No automated screening tools or machine learning–assisted screening methods were used during study selection, and no additional information was requested from study authors to determine eligibility.

### Data Collection Process

Data extraction was independently performed by 2 reviewers (YJ and KQ) using a prespecified Microsoft Excel data extraction form. Extracted information included study characteristics (author, publication year, and country), study design, participant characteristics, age, sample size, diagnostic methods for ASD, levels of VR immersion, VR devices and interaction modalities, exercise intervention type, intervention frequency, outcome measures, and primary findings. The 2 reviewers independently completed data extraction and cross-checked all extracted data for accuracy. Any disagreements arising during data extraction were resolved through discussion, with consultation from a third reviewer (KC) when necessary. When study data were missing or insufficiently reported, study authors were contacted, when feasible, to obtain additional information. No automated tools, machine learning techniques, or AI-assisted approaches were used for data extraction. Because only English-language full-text studies were included, no translation procedures were required. No specialized software was used to extract data from figures or graphs. When multiple reports originated from the same study, relevant data were combined and extracted as a single study. In cases of overlapping or inconsistent information across reports, priority was given to the report containing the most comprehensive data or the longest follow-up period.

### Data Items

This systematic review focused on the following 3 core outcome domains:

1. Behavioral outcomes, including social interaction and stereotyped repetitive behaviors, were primarily assessed using instruments such as the Social Responsiveness Scale–Second Edition (SRS-2) and the Repetitive Behaviors Scale–Revised (RBS-R).
2. Executive function outcomes included cognitive flexibility, inhibitory control, and working memory and were assessed using tasks or scales such as the Wisconsin Card Sorting Test (WCST), Flanker task, Stroop task, NIH Toolbox Dimensional Change Card Sort Test (DCCS), backward digit span task, and computerized cognitive testing program (CoSAS-S).
3. Motor outcomes included gross motor skills, motor coordination, balance performance, and overall motor function and were primarily evaluated using instruments such as the Test of Gross Motor Development–Second/Third Edition (TGMD-2/3), Movement Assessment Battery for Children–Second Edition (MABC-2), Bruininks-Oseretsky Test of Motor Proficiency–Second Edition (BOT-2), Peabody Developmental Motor Scales–Second Edition (PDMS-2), Biodex Balance System, and Motor Difficulty Scale (MDS).

For each outcome domain, all eligible measurement instruments, assessment time points, and available statistical results were extracted. In the meta-analysis, postintervention outcome data were preferentially extracted. When multiple postintervention assessment time points were reported, data collected immediately after the intervention were prioritized for quantitative synthesis, whereas results from other time points were summarized descriptively. When multiple measurement instruments were used for the same outcome, the instrument with the most complete reporting, the broadest use, or the greatest relevance to the outcome domain was prioritized for analysis. No modifications were made to the predefined outcome domain definitions or outcome selection criteria during the review process. When relevant data were missing, unclear, or unverifiable, they were recorded as “not reported,” and no assumptions or estimations were made.

### Study Risk of Bias Assessment

Two reviewers (YJ and KQ) independently assessed the risk of bias of the included studies using Cochrane Collaboration tools, applying Risk of Bias 2 (RoB 2.0; Cochrane Bias Methods Group) for RCTs and Risk of Bias in Nonrandomized Studies of Interventions (ROBINS-I; Cochrane Bias Methods Group) for non-RCTs. Any discrepancies arising during the risk-of-bias assessment were resolved through consultation with a third reviewer (KC). Under RoB 2.0, 5 domains were evaluated, including the randomization process, deviations from intended interventions, missing outcome data, outcome measurement, and selective reporting. Each domain was rated as having low risk, high risk, or some concerns, in accordance with established criteria [31].

The ROBINS-I tool assesses 7 domains of potential bias in nonrandomized studies. For the first 3 domains—bias due to confounding, participant selection, and intervention classification—the risk-of-bias assessment in nonrandomized studies differs substantially from that in randomized trials, primarily due to the absence of randomization. In contrast, for the remaining 4 domains—deviations from intended interventions, missing data, outcome measurement, and selective reporting—there is substantial overlap between the 2 assessment tools [32]. Accordingly, RoB 2.0 was applied to all included studies, with ROBINS-I additionally used to assess the first 3 domains in non-RCTs. No modifications were made to the RoB 2 or ROBINS-I tools, and no automated tools, machine learning techniques, or AI-assisted methods were used in the risk-of-bias assessment. No additional information was requested from study authors during the risk-of-bias assessment process. The results of the risk-of-bias assessment were used to inform interpretation of the findings and the overall evaluation of evidence certainty.

### Data Analysis

#### Effect Measures

All outcomes included in this systematic review were continuous variables. Because the included studies used different measurement instruments and scoring systems across behavioral, executive function, and motor outcome domains, Hedges *g* standardized mean differences (SMDs) were used as the effect size metric to improve comparability across studies and reduce potential small-sample bias. All effect sizes were reported with corresponding 95% CIs to reflect uncertainty surrounding the pooled effect estimates. Interpretation of effect size magnitude followed Cohen's conventional criteria, with 0.2 indicating a small effect, 0.5 a moderate effect, and ≥0.8 a large effect. Given the substantial variability in measurement instruments and scoring ranges across studies, pooled results were not converted into alternative effect metrics, and absolute effect estimates were not calculated.

#### Synthesis Methods

Studies were synthesized according to predefined outcome domains, including behavioral, executive function, and motor outcomes. Studies were included in the quantitative synthesis (meta-analysis) when intervention characteristics, control conditions, and outcome constructs were sufficiently comparable and postintervention data were available. Because behavioral outcomes were reported in only 3 studies and substantial differences existed in measurement instruments and assessment approaches, these outcomes were considered unsuitable for meta-analysis and were synthesized narratively. To avoid unit-of-analysis errors and double counting, each participant sample was included only once in each meta-analysis. When multiple publications reported results from the same trial, they were treated as a single study, and the data were integrated accordingly. When multiple outcome measures within the same outcome domain were reported in a single study, effect sizes were either combined or the most representative measure was selected to ensure statistical independence of effect estimates within each meta-analysis.

During data processing, postintervention means and standard deviations were preferentially extracted. To ensure consistency in effect direction, all scales were coded such that positive effect sizes consistently indicated postintervention improvement. When key statistical data were missing, study authors were contacted, when feasible, to obtain the required information. Studies with persistently incomplete data were excluded from the quantitative synthesis for the corresponding outcome. Given the anticipated clinical and methodological heterogeneity across studies in participant characteristics, VR-based exercise intervention modalities, intervention duration, and outcome measurement instruments, random-effects models were used for the meta-analysis. Effect sizes were expressed as Hedges *g* SMDs with corresponding 95% CIs and were pooled using the inverse-variance method. Between-study variance (*τ*²) was estimated using the restricted maximum likelihood (REML) method. CIs for pooled effects were adjusted using the Hartung-Knapp-Sidik-Jonkman (HKSJ) approach to reduce the risk of false-positive findings associated with the conventional DerSimonian-Laird method [33].

Statistical heterogeneity was assessed using Cochran Q tests and the *τ*², τ, and *I*² statistics. Because *I*² may not adequately capture variability in true effects across study contexts, 95% prediction intervals were additionally calculated to estimate the range within which true effects may be expected in future studies [34,35]. Given the limited number of studies included in some meta-analyses, prediction intervals were interpreted cautiously. Individual study findings and pooled effects were visualized using forest plots and presented according to outcome domains. Owing to the limited number of studies and substantial between-study variability for certain outcomes, no subgroup or sensitivity analyses were conducted. All statistical analyses were conducted using R (R Foundation for Statistical Computing), and meta-analyses were performed using the meta package.

#### Reporting Bias Assessment

Funnel plots were initially planned to assess small-study effects and potential reporting bias. Formal assessment of funnel plot asymmetry was planned when at least 10 studies were available for a given outcome. However, because fewer than 10 studies were available for both executive function and motor outcomes, and because funnel plots and related statistical tests have limited power under small-sample conditions, no formal assessment was conducted. This limitation was acknowledged in the interpretation of the findings. Egger regression tests were not performed. Funnel plot asymmetry primarily reflects small-study effects rather than publication bias alone because between-study heterogeneity, methodological differences, and random error may also contribute to asymmetry [36]. No automated tools were used, and no additional information was requested from study authors for this assessment.

#### Certainty Assessment

Two reviewers (YJ and KQ) independently evaluated the quality of evidence for each outcome using the Grading of Recommendations, Assessment, Development, and Evaluation (GRADE) framework [37]. According to GRADE criteria, the quality of evidence was classified into 4 levels, namely high, moderate, low, and very low.

The assessment of evidence quality considered study design, risk of bias, result consistency, sample size, missing data, and publication bias. Under this classification, high-quality evidence reflects rigorous study design, consistent findings, and reliable conclusions; moderate-quality evidence indicates some limitations but acceptable confidence in the results; low-quality evidence suggests potential bias or substantial heterogeneity with considerable uncertainty; and very low-quality evidence indicates serious methodological limitations and unreliable conclusions [38]. By applying the GRADE framework, the quality of evidence across all outcomes was rigorously evaluated, thereby strengthening the basis for the final conclusions.

### Ethical Considerations

Ethical approval was not required for this systematic review. This systematic review was a systematic review and meta-analysis of previously published studies and did not involve the collection of individual-level data.

## Results

### Study Selection

**Figure 1.**
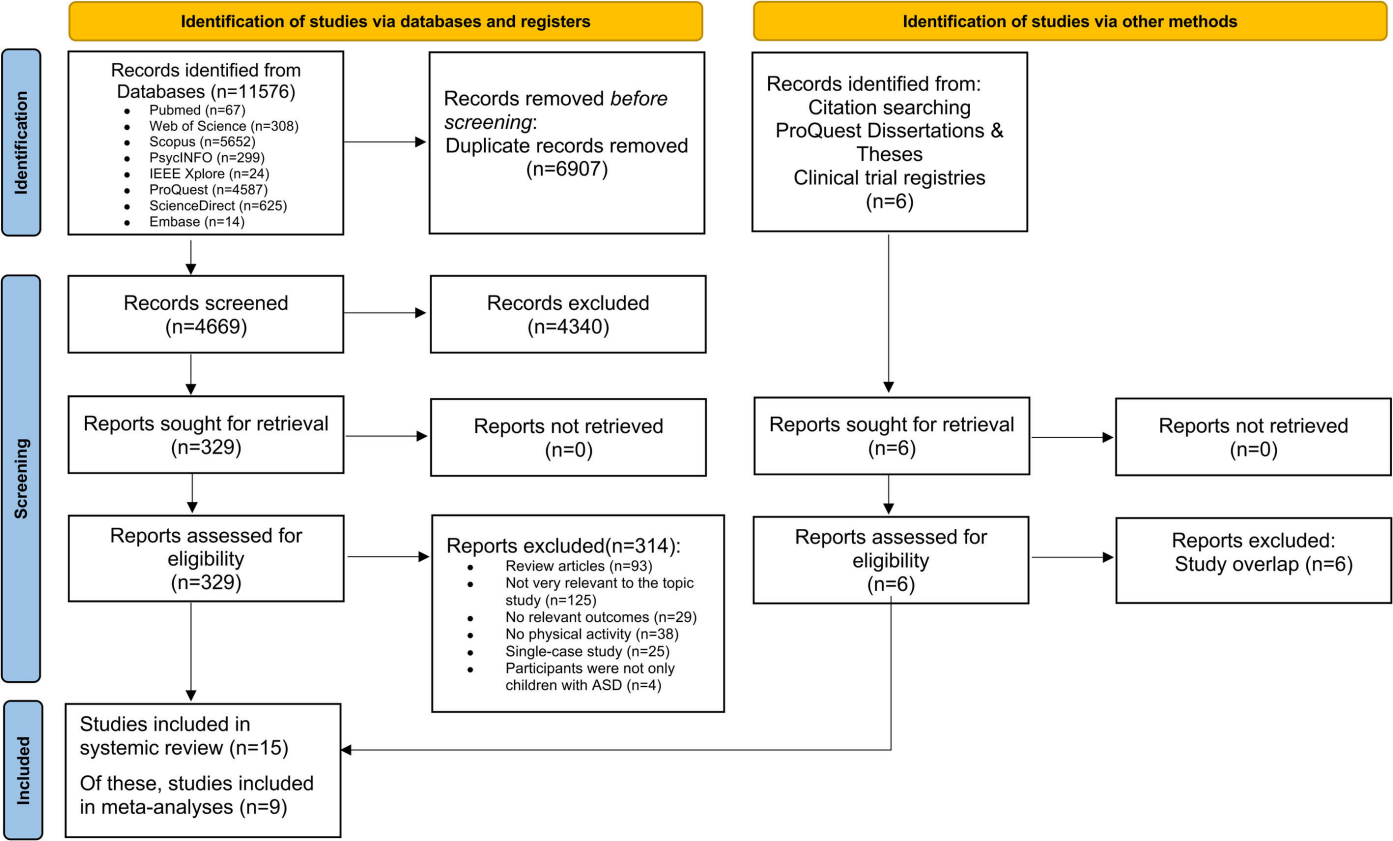
PRISMA (Preferred Reporting Items for Systematic Reviews and Meta-Analyses) flowchart.

The study selection process is presented in the PRISMA flow diagram (Figure 1). A total of 11,576 records were identified through database searching, and 4669 records remained after duplicate removal for title and abstract screening. Following title and abstract screening, 329 full-text articles were assessed for eligibility. During full-text screening, 314 articles were excluded for the following reasons: review articles (n=93), insufficient relevance to the research topic (n=125), failure to report relevant outcomes (n=29), interventions not involving physical activity (n=38), single-case study designs (n=25), and participant samples not exclusively comprising children and adolescents with ASD (n=4). An additional 6 reports were identified through other sources. However, these reports overlapped with already included studies and were therefore not treated as independent studies. Ultimately, 15 [1,2,4-8,10,39-45] studies were included in the systematic review. Of these studies, 4 used [1,6,39,45] single-group pre-post designs, 1 [4] did not provide sufficient quantitative data for effect size calculation (eg, means and SDs), and 1 [45], although reporting quantitative data, was not included in the meta-analysis because behavioral outcomes were reported in only a small number of studies and substantial heterogeneity existed across measurement methods. Therefore, these studies were included only in the narrative synthesis. In total, 9 studies [2,5,7,8,10,40-43] were included in the meta-analysis. Details of the study selection process are shown in Figure 1.

### Study Characteristics

This systematic review included 15 eligible studies, with detailed study characteristics summarized in Tables 1 and 2. Across these studies, 439 children and adolescents aged 6‐18 years with ASD were included. ASD was diagnosed using the *DSM-5* (*Diagnostic and Statistical Manual of Mental Disorders* [Fifth Edition]) in 6 [1,5,7,8,41,42] studies, the Autism Diagnostic Observation Schedule–Second Edition (ADOS-2) in 2 [10,43] studies, and the World Health Organization (WHO)’s *ICD-10* (*International Statistical Classification of Diseases, Tenth Revision*) in one [4] study. In 6 [2,6,39,40,44,45] studies, ASD diagnoses were established by qualified physicians.

**Table 1.** Study characteristics, including design, population, age, sample size, diagnostic methods, and outcome measures.

Study	Country	Study design	Population	Age (years)	Sample size (IG^a^/CG^b^)	Diagnostic methods	Outcome measures
Edwards et al (2017) [1]	Australia	Non-RCT^c^	Children and adolescents with ASD^d^	6‐10	11	DSM-5^e^	TGMD-3^f^
Vukićević et al (2019) [8]	Serbia	RCT^g^	Children and adolescents with ASD	9‐13	10 (5/5)	DSM-5	PDMS-2^h^
Rafiei Milajerdi et al (2021) [10]	Iran	RCT	Children and adolescents with ASD	6‐10	60 (40/20)	ADOS-2^i^	MABC-2^j^, WCST^k^
Hocking et al (2022) [6]	Australia	Non-RCT	Children and adolescents with ASD	10‐17	10	NR^l^	BOT-2^m^, SRS-2^n^, DCCS^o^
Ji et al (2022) [4]	China	RCT	Children and adolescents with ASD	8‐16	100 (67/34)	*ICD-10* ^p^	Flanker, Stroop
Nekar et al (2022) [39]	Korea	Non-RCT	Children and adolescents with ASD	6‐12	14	NR	SRS-2, CoSAS-S^q^
Nekar et al (2022) [2]	Korea	RCT	Children and adolescents with ASD	6‐18	24 (12/12)	NR	RBS^r^, WCST, Stroop
Lee and Jin (2023) [40]	Korea	RCT	Children and adolescents with ASD	7‐12	23 (12/11)	NR	TGMD-3
Sepehri Bonab et al (2024) [5]	Iran	RCT	Children and adolescents with ASD	7‐10	40 (2020)	DSM-5	WCST, Flanker
Miranda et al (2025) [41]	Brazil	RCT	Children and adolescents with ASD	6‐11	9 (6/3)	DSM-5	Flanker
Abdel Ghafar et al (2025) [42]	Saudi Arabia	RCT	Children and adolescents with ASD	7‐12	54 (27/27)	DSM-5	Biodex Balance System
Wu et al, 2025 [43]	China	RCT	Children and adolescents with ASD	6‐12	40 (20/20)	ADOS-2	TGMD-2^f^
Falivene et al (2025) [7]	Italy	RCT	Children and adolescents with ASD	6‐13	20 (10/10)	DSM-5	Reactive Postural Balance
Ma and Song (2025) [44]	China	RCT	Children and adolescents with ASD	8‐13	19 (9/10)	NR	SRS-2
Oliveira Neves et al (2025) [45]	Brazil	Non-RCT	Children and adolescents with ASD	7‐11	5	NR	MDS^s^

a IG: intervention group.

b CG: control group.

c Non-RCT: nonrandomized controlled trial.

d ASD: autism spectrum disorder.

e *DSM-5: Diagnostic and Statistical Manual of Mental Disorders *(Fifth Edition)*.*

f TGMD-2/3: Test of Gross Motor Development-2/3.

g RCT: randomized controlled trial.

h PDMS-2: Peabody Developmental Motor Scales, second edition.

i ADOS-2: Autism Diagnostic Observation Schedule 2.

j MABC-2: Movement Assessment Battery for Children-Second Edition.

k WCST: Wisconsin Card Sorting Test.

l NR: not reported.

m BOT-2: Bruininks-Oseretsky Test of Motor Proficiency-Second Edition.

n SRS-2: Social Responsiveness Scale‐2.

o DCCS: NIH toolbox Dimensional Change Card Sort Test.

p *ICD-10: International Classification of Diseases, Tenth Revision.*

q CoSAS-S: Computerized cognitive testing program.

r RBS: Repetitive Behaviors Scale.

s MDS: Motor Development Scale.

**Table 2.** Intervention characteristics, including virtual reality system immersive level, delivery device, mode, exercise type, frequency of intervention, and main findings.

Study	Immersive level	VR^a^ delivery device and mode	Exercise type	Frequency	Main findings
Edwards et al (2017) [1]	Semi-immersive	Xbox 360 Kinect (Microsoft), simulation-based	Sports games (tennis, golf, and bowling)	45‐60 min/d×3d/wk.×2wk	AVG^b^ can enhance individuals’ perceived skill competence and increase motivation to engage in physical activity.
Vukićević et al (2019) [8]	Semi-immersive	Xbox 360 Kinect, gamified	Tennis games	20 min/d×4d×5wk	Kinect-based educational games have a positive effect on the improvement of motor skills.
Rafiei Milajerdi et al (2021) [10]	Semi-immersive	Xbox 360 Kinect, simulation-based	Tennis games	35 min/d×3d/wk.×8wk	Exergaming effectively enhances executive function.
Hocking et al (2022) [6]	Fully immersive	HMD^c^, gamified	Dance games	20 min/d×3d/wk.×2wk	VR-based exercise interventions show no significant effects on gross motor skills or cognitive flexibility.
Ji et al (2022) [4]	Nonimmersive	Xbox 360, simple interactive	Football game	60 min/d×3d/wk.×6wk	VR training effectively improves executive function.
Nekar et al (2022) [39]	Semi-immersive	Xbox 360 Kinect, simulation-based	Cognitive and motor tasks	15 min/d×2d/wk.×3wk	Multiplayer game-based dual-task training using augmented reality (AR) significantly improves social skills and cognitive function.
Nekar et al (2022) [2]	Semi-immersive	Xbox 360 Kinect, gamified	Cognitive–motor training	15 min/d×2d/wk.×4wk	AR game-based cognitive–motor training markedly improves executive function and reaction time.
Lee and Jin (2023) [40]	Fully immersive	HMD, simulation-based	Cycling-based exergames	40 min/d×2d/wk.×12wk	VR exergames effectively enhance motor skills.
Sepehri Bonab et al (2024) [5]	Semi-immersive	Xbox 360 Kinect, simulation-based	Ball sports games	30 min/d×2d/wk.×8wk	VR-based physical exercise effectively improves executive function.
Miranda et al (2025) [41]	Semi-immersive	Xbox 360 Kinect, gamified	Dance games	20 min/d×3d	VR exergames significantly enhance inhibitory control.
Abdel Ghafar et al (2025) [42]	Semi-immersive	Nintendo Wii Fit Plus, gamified	Various sports games	30 min/d×3d/wk.×12wk	VR training effectively improves postural control.
Wu et al (2025) [43]	Semi-immersive	CAVE^d^ (Electronic Visualization Laboratory), gamified	Exergames	45 min/d×3d/wk.×12wk	VR-based serious games effectively enhance gross motor skills.
Falivene et al (2025) [7]	Semi-immersive	CAVE, gamified	Balance-training–based exergames	45 min/d×2d/wk.×5wk	VR-based postural balance training leads to modest improvements in postural balance.
Ma and Song (2025) [44]	Fully immersive	HMD, gamified	Cycling-based exergames	300 min/d×4d/wk.×16wk	VR motion-based serious games effectively enhance social interaction skills.
Oliveira Neves et al (2025) [45]	Nonimmersive	Nintendo Wii Fit Plus, gamified	Various sports games	-min/d×2d/wk.×12wk	VR exergames significantly improve motor skills.

a VR: virtual reality.

b AVG: active video game.

c HMD: head-mounted display.

d CAVE: cave automatic virtual environment.

Of the 15 studies, 3 were conducted in China, 3 in Korea, 2 each in Australia, Brazil, and Iran, and one each in Serbia, Saudi Arabia, and Italy. Regarding study design, 11 studies were RCTs, whereas 4 used single-group pre-post designs.

Regarding intervention modalities, 8 studies used Xbox 360 Kinect (Microsoft)–based systems, 3 used head-mounted displays (HMDs), 2 used cave automatic virtual environment (CAVE; Electronic Visualization Laboratory), and 2 used the Nintendo Wii Fit Plus (Nintendo). The physical activity interventions primarily consisted of sport-based games, including tennis, golf, bowling, soccer, cycling, dancing, and rugby. Intervention duration ranged from 3 days to 16 weeks across the included studies.

For behavioral outcomes, social communication was assessed using the SRS-2 in 3 studies, while repetitive and stereotyped behaviors were measured using the Repetitive Behavior Scale–Revised in one study. Executive function was assessed using the WCST (3 studies), the Flanker Task (3 studies), the Stroop Color–Word Test (2 studies), the DCCS (one study), and the CoSAS-S (one study). Motor abilities were evaluated using the TGMD-2/3 (2 studies), the MABC-2 (2 studies), the BOT-2 (2 studies), the PDMS-2 (1 study), the Biodex Balance System (1 study), the Reactive Postural Balance (1 study), and the MDS (1 study).

### Risk of Bias Assessment

**Figure 2.**
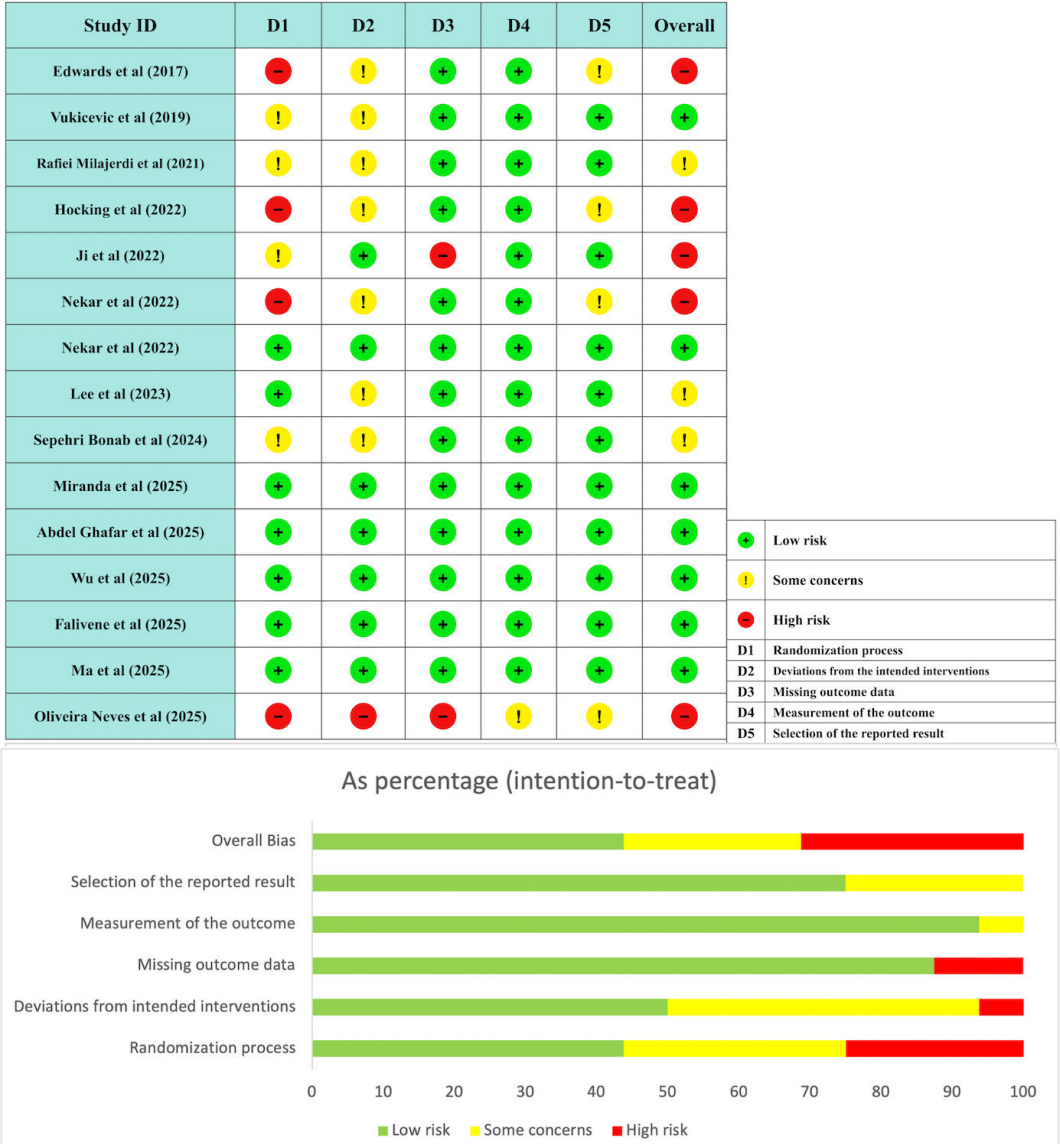
Summary of the risk of bias of the included studies (Risk of Bias 2 [RoB 2.0]) [1,2,4-8,10,39-45].

**Figure 3.**
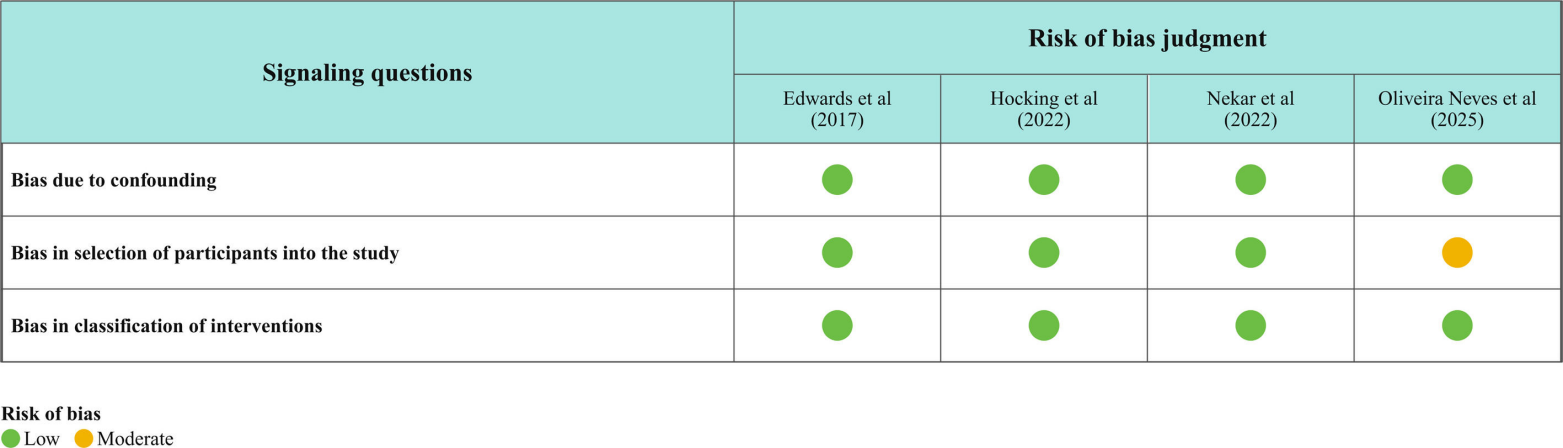
Bias judgment of nonrandomized comparison studies (Risk of Bias in Nonrandomized Studies of Interventions [ROBINS-I]) [1,2,6,45].

Figure 2 [1,2,4-8,10,39-45] and Figure 3 [1,2,6,45] summarize the risk-of-bias assessment for all included studies. Across the 5 domains, 7 studies were judged to be at low risk of bias, 3 raised some concerns, and 5 were rated as high risk, primarily due to deficiencies in the randomization process in nonrandomized studies and the lack of reported means and SDs for outcome data in several studies. Overall, the included studies showed a relatively low risk of bias in the domains of deviations from intended interventions and outcome measurement. For the 4 nonrandomized controlled studies, 3 domains related to the absence of randomization were additionally assessed. Of the 12 judgments assessed in these domains, one was rated as moderate risk, whereas the remaining judgments were classified as low risk.

### Results of Individual Studies and Syntheses

#### Behavioral Outcomes

Among the 15 included studies, only 3 examined the effects of VR-based physical exercise interventions on behavioral outcomes in children and adolescents with ASD, indicating that evidence in this domain remains limited. Behavioral outcomes were primarily assessed using the SRS-2 and the Repetitive Behavior Scale–Revised, which measure social interaction difficulties and repetitive, stereotyped behaviors, respectively. Of these studies, 1 used a single-group pre-post design, whereas the remaining 2 were RCTs. Given the limited number of studies and substantial heterogeneity across study designs, behavioral outcome domains, and measurement instruments, a meta-analysis was not performed, and the findings were synthesized narratively.

As summarized in Tables 1 and 2, one study using an augmented reality (AR)–based system that integrated cognitive tasks with motor training reported significant improvements in social interaction scores in children with ASD compared with baseline [39]. However, the absence of a control group limits the interpretability of these findings, as they primarily reflect within-group pre-post changes and therefore represent a low level of evidence.

In RCTs, the effects of VR-based physical exercise interventions on behavioral outcomes were inconsistent. One RCT evaluated an interactive VR-based motion-serious game for children with ASD and reported significant postintervention improvements in social interaction behaviors in the experimental group compared with baseline [44]. In contrast, another RCT reported significant reductions in repetitive and stereotyped behaviors in both intervention and control groups, with no significant between-group differences, suggesting that the intervention-specific effects remain unclear [2].

Overall, current evidence regarding the effectiveness of VR-based exercise interventions for improving behavioral outcomes in children and adolescents with ASD remains limited. Because of the limited number of available studies, small sample sizes, and substantial heterogeneity in study designs and behavioral outcome measures, no definitive conclusions can currently be drawn. Additional high-quality RCTs are warranted to further validate these findings.

#### Executive Function

Among the 15 included studies, 7 examined the effects of VR-based physical exercise interventions on executive function in children and adolescents with ASD. These studies primarily targeted core components of executive function, including inhibitory control, working memory, cognitive flexibility, and attention, assessed using behavioral tasks and computerized tests. Of these studies, 3 were included only in the systematic review, whereas 4 met the criteria for inclusion in the meta-analysis.

#### Systematic Review

As summarized in Tables 1 and 2, single-group pre-post studies showed heterogeneous postintervention changes across executive function domains. One study using a fully immersive VR-based exercise game found no significant postintervention improvement in cognitive flexibility among adolescents with ASD [6]. In contrast, another study using AR-based cognitive-motor training reported significant postintervention improvements across multiple cognitive domains, including orientation, memory, attention, and visual perception, in children with ASD. However, the absence of control groups limits the interpretability of these findings, as they primarily reflect within-group pre-post changes and therefore represent a low level of evidence [39].

In RCTs, most studies demonstrated positive effects of VR-based physical exercise interventions on executive function. Several studies showed that exercise interventions delivered via motion-sensing platforms or VR environments significantly improved overall executive function in children with ASD following the intervention [4,5,10]. Another RCT using AR-based cognitive-motor games reported significantly greater improvements in executive function–related outcomes in the intervention group than in the control group [2]. One study found that exergames produced significantly greater improvements in inhibitory control in children with ASD [41].

Available evidence suggests that VR-based physical exercise interventions have substantial potential to improve executive function in children and adolescents with ASD. However, heterogeneity in intervention modalities, assessment tools, and the executive function subcomponents targeted may limit the comparability of findings across studies.

#### Meta-Analysis

**Figure 4.**
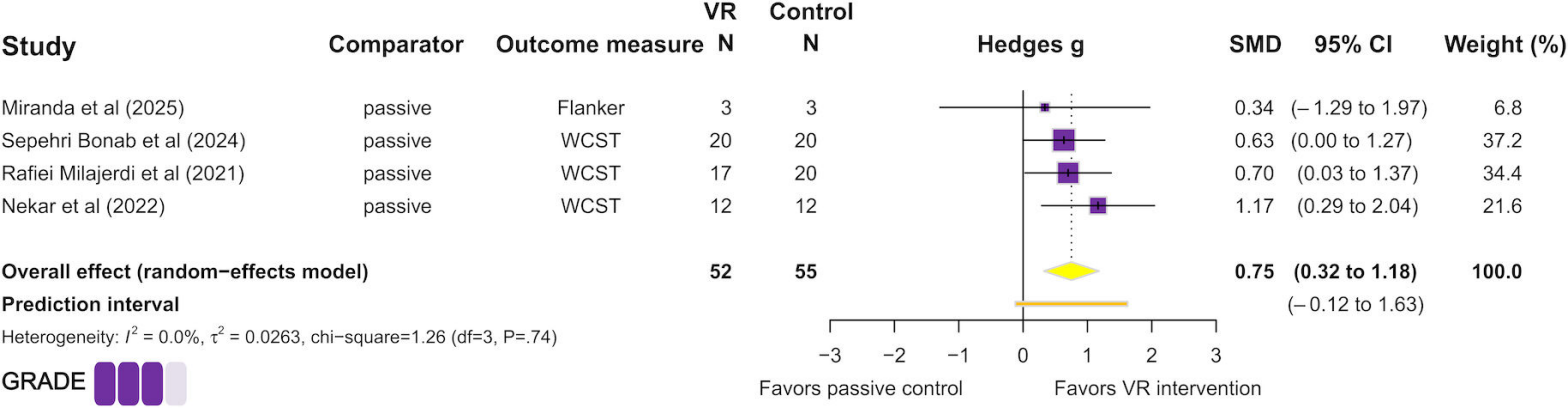
Meta-analysis of virtual reality (VR)–based physical exercise interventions on executive function in children and adolescents with autism spectrum disorder (ASD) [2,5,10,41]. GRADE: Grading of Recommendations, Assessment, Development, and Evaluation; SMD: standardized mean difference; VR: virtual reality; WCST: Wisconsin Card Sorting Test.

A total of 4 RCTs involving 107 children and adolescents with ASD were included in the meta-analysis. As shown in Figure 4 [2,5,10,41], VR-based exercise interventions demonstrated a moderate pooled effect on executive function outcomes (SMD 0.75, 95% CI 0.32-1.18; *P*=.01). Statistical heterogeneity across studies was low (*I*²=0.0%; *τ*²=0.0263; *P*=.74). To further evaluate the distribution of true effects across study contexts, 95% prediction intervals were additionally calculated. The prediction interval ranged from −0.12 to 1.63. The wide prediction interval suggested substantial uncertainty across study settings. Although the pooled effect supported the potential benefits of VR-based exercise interventions for executive function, the prediction interval crossed the null effect line, suggesting that intervention effects may vary across study settings. According to the GRADE framework, the certainty of evidence for this outcome was rated as moderate. However, given the relatively small sample sizes and methodological limitations of some studies, the current evidence should be interpreted cautiously, and further high-quality, large-scale RCTs are needed to confirm these findings.

#### Motor Outcomes

Of the 15 included studies, 9 examined the effects of VR-based physical exercise interventions on motor performance in children and adolescents with ASD. These studies primarily assessed core components of motor performance, including fine motor skills, gross motor skills, and balance, using assessment tools such as the MABC-2, BOT-2, and TGMD-3. Of these studies, 3 were included only in the systematic review, whereas 6 were eligible for meta-analysis.

#### Systematic Review

As shown in Tables 1 and 2, single-group pre-post studies demonstrated inconsistent effects of VR-based physical exercise interventions on motor performance in children and adolescents with ASD. One study using AVGs suggested that exergames, when used as the sole intervention modality, may not provide sufficient opportunities for repeated practice of normative movement patterns, thereby limiting direct improvements in motor skills. However, the study also found that such interventions may enhance children’s perceived motor competence and, to some extent, increase motivation to engage in physical activity [1]. Another study using fully immersive VR-based exercise games did not detect significant postintervention improvements in gross motor skills among adolescents with ASD [6]. Because these studies lacked control groups, their findings primarily reflect within-group pre–post changes, resulting in a relatively low level of evidence.

In RCTs, the effects of VR-based physical exercise interventions on motor outcomes were more heterogeneous. Several studies reported that exercise interventions delivered in AR or VR environments significantly improved overall motor skills, balance, or object control in children with ASD, with intervention groups outperforming control groups on selected outcomes [7,8,43,45]. In addition, some studies indicated that incorporating VR training as an adjunct to conventional physical exercise or physiotherapy yielded additional benefits in postural control or balance [40,42]. Conversely, some RCTs did not observe significant advantages of VR-based exercise interventions in motor skills or physical activity levels [10,18].

Substantial between-study differences in VR immersion level, intervention content, training dosage, and motor outcome measures may partially undermine the comparability and stability of the findings.

#### Meta-Analysis

**Figure 5.**
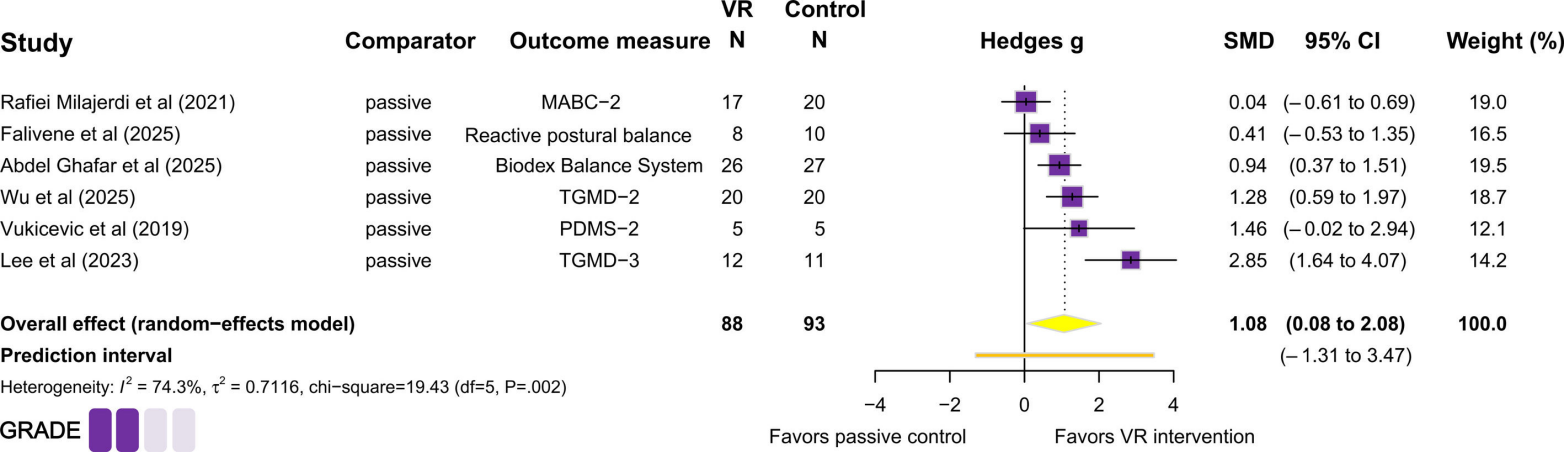
Meta-analysis of virtual reality (VR)–based physical exercise interventions on motor outcomes in children and adolescents with autism spectrum disorder (ASD) [7,8,10,40,42,43]. GRADE: Grading of Recommendations, Assessment, Development, and Evaluation; SMD: standardized mean difference; MABC-2: Movement Assessment Battery for Children–Second Edition; PDMS-2: Peabody Developmental Motor Scales–Second Edition; TGMD-2: Test of Gross Motor Development–Second Edition; VR: virtual reality.

A total of 6 RCTs involving 181 children and adolescents with ASD were included in the meta-analysis. As shown in Figure 5 [7,8,10,40,42,43], VR-based exercise interventions demonstrated a large pooled effect on motor outcomes (SMD 1.08, 95% CI 0.08-2.08; *P*=.04). However, substantial between-study heterogeneity was observed (*I*²=74.3%; *τ*²=0.7116; *P*=.001), indicating considerable variability across study findings. To further evaluate the potential distribution of true effects across study contexts, 95% prediction intervals were additionally calculated. The prediction interval ranged from −1.31 to 3.47, suggesting that the true effect in future studies may range from no meaningful benefit or even negative effects to substantial improvement. According to the GRADE framework, the certainty of evidence for this outcome was rated as low, primarily because of substantial heterogeneity, small sample sizes, and methodological limitations of some studies. Therefore, although the pooled effect suggests that VR-based exercise interventions may improve motor outcomes in children and adolescents with ASD, the stability and certainty of the current evidence remain limited, and the findings should be interpreted cautiously.

### Certainty of Evidence

According to the GRADE framework, the certainty of evidence differed across outcome domains (Table 3). The certainty of evidence for motor performance outcomes was rated as low, mainly due to considerable heterogeneity and moderate imprecision. In contrast, the certainty of evidence for executive function outcomes was rated as moderate and was downgraded solely due to concerns regarding risk of bias in some studies. Overall, although VR-based physical exercise interventions showed relatively consistent beneficial effects on executive function, the strength of evidence supporting behavioral and motor outcomes remains limited. Detailed GRADE assessment procedures and the specific reasons for downgrading each outcome are provided in the Summary of Findings table (S2 in Multimedia Appendix 1).

**Table 3.** Grading of Recommendations, Assessment, Development, and Evaluation (GRADE) summary of findings table.

Certainty assessment							Number of patients		Effect (95% CI)	Certainty
Number of studies	Study design	Risk of bias	Inconsistency	Indirectness	Imprecision	Other considerations	VR^a^	Control		
Executive function										
4	Randomized trials	Not serious	Not serious	Not serious	Serious^b^	None	52	55	SMD^c^ 0.75 SD higher (0.32 higher to 1.18 higher)	⨁⨁⨁◯^d^ Moderate^b^
Motor outcomes										
6	Randomized trials	Not serious	Serious^e^	Not serious	Serious^b^	None	88	93	SMD 1.08 SD higher (0.08 higher to 2.08 higher)	⨁⨁◯◯ Low^b,e^

a VR: virtual reality.

b Sample size too small

c SMD: standardized mean difference.

d ⊕⊕⊕⊕ High certainty; ⊕⊕⊕○ Moderate certainty; ⊕⊕○○ Low certainty; ⊕○○○ Very low certainty.

e *I*² > >70%

## Discussion

### Principal Findings

This study systematically reviewed and meta-analyzed the effects of VR–based exercise interventions on behavioral outcomes, executive function, and motor performance in children and adolescents with ASD. Unlike previous reviews that primarily focused on general VR training [28], social skills interventions [46], or single functional domains [47], this review specifically examined VR interventions centered on physical activity participation and integrated behavioral, executive function, and motor outcomes to better clarify the potential clinical value of VR-based exercise interventions within comprehensive ASD intervention frameworks.

Overall, the findings indicate distinct evidence patterns across outcome domains. Current evidence suggests that findings for behavioral outcomes remain inconsistent, whereas executive function outcomes demonstrate relatively consistent positive effects and motor outcomes exhibit substantial between-study variability and contextual dependence. Moreover, certainty of evidence, heterogeneity, and risk of bias differed substantially across outcome domains. Therefore, interpretation of intervention effectiveness should emphasize evidence consistency and contextual applicability rather than relying solely on pooled effect estimates.

In addition, the VR interventions included in this review exhibited substantial conceptual heterogeneity, encompassing AVGs [18], motion-based interactive training [43], AR-based exercise training [2], and VR systems with varying levels of immersion [44]. Therefore, future research should more precisely differentiate interventions according to immersion level, feedback modality, social interaction format, and exercise task specificity rather than pooling different VR exercise modalities into a single intervention category, thereby improving interpretability and clinical applicability.

### Effects of VR-Based Physical Exercise Interventions on Behavioral Outcomes in Children and Adolescents With ASD

This review did not identify a stable or consistent overall effect of VR-based exercise interventions on behavioral outcomes in children and adolescents with ASD. Nevertheless, several individual studies reported improvements in social interaction or repetitive stereotyped behaviors [2,39,44]. This pattern—where positive findings were reported in individual studies despite inconsistent overall evidence—suggests that behavioral outcomes may be highly context-dependent, with intervention effects potentially influenced by training content, interaction modalities, outcome measures, and study design characteristics.

Social motivation theory and mirror neuron system models suggest that interactions with virtual avatars in low-threat virtual environments may facilitate social engagement and imitative behaviors [48]. However, the VR interventions included in this review primarily emphasized physical activity participation, with intervention goals focused on exercise engagement, motor control, task completion, and cognitive processing rather than directly targeting the core social behavioral impairments associated with ASD. Consequently, behavioral improvements may depend largely on whether VR tasks incorporate training mechanisms aligned with the targeted behavioral outcomes [39]. For example, Nekar et al [39] reported that although their AR cognitive-motor training included interactive tasks, the absence of interactive verbal dialogue systems within the game environment may have limited its capacity to promote social communication and language development. The authors further suggested that improvements in social behaviors and higher-order cognitive functions may require longer-term and sustained training exposure. This interpretation is consistent with the findings of the present review, suggesting that although most VR-based exercise interventions may enhance engagement and task involvement, their social interaction components are often limited, particularly regarding real-world verbal communication, emotional feedback, and bidirectional social interaction, thereby restricting the transfer of intervention effects to real-world social behaviors.

In contrast, several studies reported improvements in repetitive stereotyped behaviors. Nekar et al [2] reported that AR-based gamified cognitive-motor training reduced purposeless movements and repetitive behaviors. The authors suggested that these improvements may be associated with the sensory stimulation and physical exertion generated through simultaneous motor and cognitive engagement. Previous studies have suggested that higher levels of physical activity may reduce stereotyped behaviors through postexercise fatigue mechanisms [49]. In addition, the visual, motor, and sensory stimulation provided by AR/VR environments may partially substitute for self-stimulatory needs, thereby decreasing the frequency of repetitive stereotyped behaviors [50]. These findings suggest that the mechanisms underlying VR-based exercise interventions may differ across behavioral outcome types. Improvements in social communication may depend more heavily on sophisticated interaction designs, whereas reductions in repetitive behaviors may be more closely related to physical activity engagement and sensory stimulation [51-55].

Therefore, the present review does not support the simplistic conclusion that VR-based exercise interventions are ineffective for behavioral outcomes. Rather, the available evidence suggests that their behavioral effects are highly task-specific and intervention-dependent. Substantial differences across VR systems in interaction modalities, feedback mechanisms, immersion levels, and integration of social elements may represent one important source of the inconsistencies observed in current behavioral outcome findings. These findings highlight a critical issue within the field: VR technology itself does not inherently produce behavioral improvement; rather, its effectiveness may depend on the specific skills and behaviors targeted within the VR environment. Future studies should further differentiate the behavioral training mechanisms embedded within different types of VR-based exercise interventions, such as verbal interaction, cooperative tasks, social feedback, multiplayer interaction, or contextual generalization training, and investigate which intervention designs are most likely to facilitate the transfer of effects to real-world social behaviors.

### Effects of VR-Based Physical Exercise Interventions on Executive Function in Children and Adolescents With ASD

Among the outcome domains included in this review, executive function demonstrated the most consistent evidence base. Both the systematic review and meta-analysis suggested that VR-based exercise interventions may positively affect multiple domains of executive function in children and adolescents with ASD, including inhibitory control, working memory, attention, and cognitive flexibility, with relatively low between-study heterogeneity [2,5,10,41].

The positive effects of VR-based exercise interventions on executive function are generally consistent with previous findings. Sepehri Bonab reported that VR-based exercise games integrate engaging task designs, real-time feedback, interactivity, and immersive experiences, thereby providing children and adolescents with ASD with training environments that simultaneously require physical participation and cognitive processing, which may facilitate executive function development [5]. Related studies further suggest that virtual exercise games may not only improve executive function performance but also promote physical health, self-perception, and participation in moderate-to-vigorous physical activity [41,56]. These interventions typically require children to process multiple stimuli across physical and virtual environments while making rapid decisions, thereby imposing substantial cognitive demands that may activate executive control–related brain networks more effectively than exercise modalities focused primarily on physical activity alone [57]. However, some studies have reported that virtual training is not consistently superior to conventional physical exercise for improving executive function, suggesting that intervention effectiveness may depend on factors such as game type, immersion level, and training content [4].

Previous research suggests that exercise may improve executive function in children and adolescents with ASD through multiple interrelated mechanisms [55]. From a neurobiological perspective, exercise may promote structural and functional neuroplasticity by enhancing the functioning of brain regions closely associated with executive processes, such as the prefrontal cortex and hippocampus, thereby supporting improvements in working memory and cognitive flexibility [51]. Furthermore, functional near-infrared spectroscopy studies have demonstrated that exercise can significantly increase prefrontal cortical activation in individuals with ASD, and this enhanced activation appears to be closely associated with improved executive task performance [58]. At the psychosocial level, physical activity may further enhance the beneficial effects of exercise interventions on executive function by promoting social participation, fostering positive experiences, and increasing motivation [52-55]. Therefore, the potential value of VR technology may lie primarily in its ability to enhance engagement and adherence through gamification, real-time feedback, and structured task design, thereby strengthening the effects of exercise interventions on executive function rather than producing independent effects through immersive technology alone.

### Effects of VR-Based Physical Exercise Interventions on Motor Outcomes in Children and Adolescents With ASD

Compared with executive function outcomes, motor outcomes demonstrated substantially greater heterogeneity and uncertainty. Although several studies reported positive trends in balance performance [7], object control skills [1], and overall motor proficiency following VR-based exercise interventions [10,40,43], substantial variability was observed across studies, and the prediction intervals suggested that intervention effects may vary considerably across different research contexts. These findings indicate that the observed pooled effects may not be generalizable across all forms of VR-based exercise interventions or all subgroups of children and adolescents with ASD.

This inconsistency may primarily reflect substantial intervention heterogeneity across included studies. The VR interventions included in this review involved various device types, including Xbox Kinect, Nintendo Wii, immersive head-mounted display systems, and AR platforms, as well as differing immersion levels and exercise training content, all of which may influence motor learning processes and intervention outcomes [59]. For example, some studies implemented structured exercise training characterized by explicit movement goals, repetitive practice, and immediate motion feedback, and these studies were generally more likely to report positive outcomes [7,8,43,45]. In contrast, other studies emphasized entertainment, free interaction, or general physical activity participation, which may have produced more limited improvements in specific motor skills while primarily enhancing training tolerance and participation motivation, both of which are considered important determinants of exercise intervention effectiveness and sustainability [8,60,61].

Additionally, differences across VR systems in feedback modalities, motion-capture precision, forms of social interaction, and sensory stimulation complexity may influence exercise participation experiences and training responses in children and adolescents with ASD [62,63]. For some children and adolescents with ASD who exhibit sensory sensitivities or attentional regulation difficulties, highly immersive environments may not necessarily produce superior training outcomes, because complex sensory stimuli may increase cognitive load or sensory stress in some individuals with ASD [28]. Accordingly, the findings of this review suggest that the effectiveness of VR-based exercise interventions may be highly context-dependent and that different intervention modalities should not be simplistically treated as homogeneous interventions.

Notably, several studies have indicated that when VR training is used as an adjunct to conventional physical activity or physical therapy, positive changes are more frequently observed in postural control, balance performance, and training engagement [40,42]. These findings suggest that VR-based exercise interventions may be more appropriately positioned as adjunctive or enhancement-based interventions within the motor domain rather than as complete replacements for traditional exercise training. In other words, their potential value may lie primarily in enhancing training enjoyment, increasing opportunities for movement repetition, and strengthening real-time feedback rather than independently providing comprehensive motor rehabilitation [4]. Based on these findings, future research should more precisely differentiate interventions according to immersion level, feedback modality, social interaction format, and exercise task specificity rather than pooling different VR exercise modalities into a single intervention category, thereby improving interpretability and clinical applicability.

### Implications for Clinical Practice and Future Research

The findings of this systematic review suggest that VR-based exercise interventions may hold promise within comprehensive intervention frameworks for children and adolescents with ASD. However, this potential should be understood primarily as adjunctive and context-dependent rather than as a direct replacement for conventional intervention approaches. From a clinical perspective, VR-based exercise interventions may be particularly suitable for children and adolescents with ASD who demonstrate low motivation for physical activity participation, heightened sensory sensitivity, or difficulty maintaining long-term engagement in traditional exercise programs [6,8,10]. Compared with conventional training approaches, the real-time feedback, gamified features, and structured task designs embedded within VR environments may improve engagement and adherence while providing some children with more predictable and lower-stress training settings [41]. However, this evidence remains insufficient to support the widespread implementation of VR-based exercise interventions as stand-alone treatments, particularly for improving core behavioral symptoms [2,39,44]. Therefore, their clinical value should be interpreted cautiously. At present, VR-based exercise interventions may be more appropriately positioned as complementary tools within conventional exercise training, rehabilitation programs, or comprehensive behavioral interventions to enhance participation and training experiences rather than replace real-world social interaction and exercise training.

This review also provides several implications for future research. First, future studies should further standardize the reporting of VR interventions, including key parameters such as immersion level, feedback format, exercise task structure, social interaction modality, and training dosage, to improve comparability across studies. Second, future research should place greater emphasis on the potential moderating effects of different VR characteristics on intervention outcomes, including differences between immersive and nonimmersive systems, individual versus socially interactive training, and structured motor learning versus entertainment-oriented activities. Third, because most existing studies focus primarily on short-term outcomes, future investigations should incorporate long-term follow-up assessments to evaluate the durability and real-world generalizability of intervention effects. In addition, substantial variability exists in the behavioral, executive function, and motor outcome measures used across studies, highlighting the need for more standardized and clinically interpretable assessment frameworks.

More importantly, future research should move beyond simply asking whether VR is effective and instead investigate which types of VR-based exercise training are most likely to produce beneficial effects, for which subgroups of children and adolescents with ASD, and under which contextual conditions. Compared with simply contrasting “VR versus non-VR” interventions, this more precise and mechanism-oriented research framework may better facilitate the clinical translation and individualized implementation of VR-based exercise interventions in the ASD field.

### Limitations

Several limitations of this systematic review should be acknowledged. First, the overall number of included studies was limited, and some outcome domains included only a small number of studies, which may have reduced the stability and precision of the pooled effect estimates. In particular, the wide CIs and low certainty of evidence for behavioral outcomes indicate substantial uncertainty in the current findings.

Second, the VR-based exercise interventions included in this review demonstrated substantial conceptual heterogeneity. Considerable variability was observed across studies in terms of VR device types, immersion levels, feedback mechanisms, training content, and implementation approaches, all of which may influence intervention effectiveness. Accordingly, pooling heterogeneous VR exercise modalities into a single intervention category may have reduced interpretability and limited conclusions regarding the effects of specific intervention models.

Third, some meta-analytic findings demonstrated substantial heterogeneity and wide prediction intervals, suggesting that intervention effects may vary considerably across research settings. This finding suggests that the observed pooled effects may not be consistently generalizable across all clinical or educational settings. Therefore, the findings of this review should be interpreted as reflecting overall trends within the current evidence base rather than definitive conclusions applicable to all forms of VR-based exercise interventions.

Furthermore, several studies were subject to risks of bias related to randomization procedures, outcome measurement, and selective reporting, while small sample sizes and short follow-up durations further reduced the certainty of the evidence. In addition, most included studies focused on children and adolescents with mild-to-moderate ASD, and evidence regarding the applicability of these interventions to individuals with more severe ASD, different age groups, and diverse cultural backgrounds remains limited.

Finally, owing to the limited number of included studies, this review was unable to comprehensively examine the potential effects of different VR characteristics, training dosages, and participant characteristics through meta-regression or detailed subgroup analyses. Consequently, interpretations regarding differences among various VR exercise modalities should be made cautiously at this stage.

### Conclusions

This systematic review systematically synthesized the current evidence regarding VR-based exercise interventions for children and adolescents with ASD. Unlike previous reviews that primarily focused on general VR training, social skills interventions, or single functional domains, this review specifically emphasized VR interventions centered on physical activity participation and simultaneously integrated behavioral, executive function, and motor outcomes, thereby providing a more comprehensive understanding of their multidimensional effects. Current evidence suggests that VR-based exercise interventions may produce relatively consistent positive effects on executive function, whereas their effects on motor and behavioral outcomes remain uncertain and highly context-dependent. Accordingly, these interventions may currently be more appropriately regarded as promising adjunctive tools rather than fully established stand-alone intervention alternatives.

Consistent with these findings, this review suggests that future VR research in the ASD field should move beyond focusing solely on whether VR is used and instead place greater emphasis on how VR interventions are designed, which goals they target, and the mechanisms through which they exert their effects. From a clinical perspective, VR-based exercise interventions may provide new opportunities to enhance training enjoyment, participation motivation, and individualized adaptation to intervention programs. From a research perspective, this review highlights the importance of developing more standardized, mechanism-oriented, and longitudinal study designs. These findings may help facilitate the transition of VR-based exercise interventions from emerging technological applications to evidence-based adjunctive intervention tools.

## Supplementary material

10.2196/98579 Multimedia Appendix 1 Search strategy and Grading of Recommendations, Assessment, Development, and Evaluation (GRADE) summary of findings table.

10.2196/98579 Checklist 1 PRISMA 2020 for abstracts checklist.

10.2196/98579 Checklist 2 PRISMA checklist.

## Abbreviations

ADOS-2: Autism Diagnostic Observation Schedule–Second Edition
AR: augmented reality
ASD: autism spectrum disorder
AVG: active video game
BOT-2: Bruininks-Oseretsky Test of Motor Proficiency–Second Edition
CAVE: cave automatic virtual environment
CoSAS-S: computerized cognitive testing program
DCCS: Dimensional Change Card Sort Test
*DSM-5*: *Diagnostic and Statistical Manual of Mental Disorders* (Fifth Edition)
GRADE: Grading of Recommendations, Assessment, Development, and Evaluation
HKSJ: Hartung-Knapp-Sidik-Jonkman
HMD: head-mounted display
*ICD-10*: *International Statistical Classification of Diseases, Tenth Revision*
MABC-2: Movement Assessment Battery for Children–Second Edition
MDS: Motor Development Scale
PDMS-2: Peabody Developmental Motor Scales–Second Edition
PICOS: Population, Intervention, Comparison, Outcomes, and Study design
PRISMA: Preferred Reporting Items for Systematic Reviews and Meta-Analysis
PROSPERO: International Prospective Register of Systematic Reviews
RBS-R: Repetitive Behaviors Scale–Revised
RCT: randomized controlled trial
REML: restricted maximum likelihood
RoB 2: Risk of Bias 2
ROBINS-I: Risk of Bias in Nonrandomized Studies of Interventions
SMD: standardized mean difference
SRS-2: Social Responsiveness Scale–Second Edition
TGMD-2/3: Test of Gross Motor Development–Second/Third Edition
VR: virtual reality
WCST: Wisconsin Card Sorting Test
WHO: World Health Organization

## References

[1] Edwards J, Jeffrey S, May T, Rinehart NJ, Barnett LM (2017). Does playing a sports active video game improve object control skills of children with autism spectrum disorder?. J Sport Health Sci.

[2] Nekar DM, Lee DY, Hong JH (2022). Effects of augmented reality game-based cognitive-motor training on restricted and repetitive behaviors and executive function in patients with autism spectrum disorder. Healthcare (Basel).

[3] Sudo M, Komiyama T, Aoyagi R, Nagamatsu T, Higaki Y, Ando S (2017). Executive function after exhaustive exercise. Eur J Appl Physiol.

[4] Ji C, Yang J, Lin L, Chen S (2022). Executive function improvement for children with autism spectrum disorder: a comparative study between virtual training and physical exercise methods. Children (Basel).

[5] Sepehri Bonab H, Ebrahimi Sani S, Behzadnia B (2025). The impact of virtual reality intervention on emotion regulation and executive functions in autistic children. Games Health J.

[6] Hocking DR, Ardalan A, Abu-Rayya HM (2022). Feasibility of a virtual reality-based exercise intervention and low-cost motion tracking method for estimation of motor proficiency in youth with autism spectrum disorder. J Neuroeng Rehabil.

[7] Falivene A, Scaccabarozzi G, Busti Ceccarelli S (2025). Virtual reality-based postural balance training in autistic children: a pilot randomized controlled trial. J Clin Med.

[8] Vukićević S, Đorđević M, Glumbić N, Bogdanović Z, Đurić Jovičić M (2019). A demonstration project for the utility of kinect-based educational games to benefit motor skills of children with ASD. Percept Mot Skills.

[9] Ketcheson L, Hauck J, Ulrich D (2017). The effects of an early motor skill intervention on motor skills, levels of physical activity, and socialization in young children with autism spectrum disorder: a pilot study. Autism.

[10] Rafiei Milajerdi H, Sheikh M, Najafabadi MG, Saghaei B, Naghdi N, Dewey D (2021). The effects of physical activity and exergaming on motor skills and executive functions in children with autism spectrum disorder. Games Health J.

[11] Houghton R, van den Bergh J, Law K, Liu Y, de Vries F (2021). Risperidone versus aripiprazole fracture risk in children and adolescents with autism spectrum disorders. Autism Res.

[12] Cai KL, Wang JG, Liu ZM (2020). Mini-Basketball training program improves physical fitness and social communication in preschool children with autism spectrum disorders. J Hum Kinet.

[13] Mortimer R, Privopoulos M, Kumar S (2014). The effectiveness of hydrotherapy in the treatment of social and behavioral aspects of children with autism spectrum disorders: a systematic review. J Multidiscip Healthc.

[14] Martínez Moreno CM, Hernández Garre JM, Echevarría Pérez P, Morales Moreno I, Vegue Parra E, Valero Merlos E (2025). Effectiveness of equine-assisted intervention as a therapeutic strategy for improving adaptive behaviour in children with autism spectrum disorder. Healthcare (Basel).

[15] Watters RG, Watters WE (1980). Decreasing self-stimulatory behavior with physical exercise in a group of autistic boys. J Autism Dev Disord.

[16] Zhang L, Zhang C, Yuan X, Ji Y (2025). The impact of exercise interventions on core symptoms of 3-12-year-old children with autism spectrum disorder: a systematic review and network meta-analysis. Eur Child Adolesc Psychiatry.

[17] Gao X, Xu G, Fu N (2025). Exercise interventions for health outcomes in children with autism spectrum disorder: an umbrella review of meta-analyses of clinical trials. Neurosci Biobehav Rev.

[18] Lau PWC, Wang G, Wang JJ (2020). Effectiveness of active video game usage on body composition, physical activity level and motor proficiency in children with intellectual disability. J Appl Res Intellect Disabil.

[19] Pennington DL, Reavis JV, Cano MT, Walker E, Batki SL (2022). The impact of exercise and virtual reality executive function training on cognition among heavy drinking veterans With traumatic brain injury: a pilot feasibility study. Front Behav Neurosci.

[20] Bellani M, Fornasari L, Chittaro L, Brambilla P (2011). Virtual reality in autism: state of the art. Epidemiol Psychiatr Sci.

[21] Malihi M, Nguyen J, Cardy RE, Eldon S, Petta C, Kushki A (2020). Data-driven discovery of predictors of virtual reality safety and sense of presence for children with autism spectrum disorder: a pilot study. Front Psychiatry.

[22] Yazdanian H, Vakili A, Soltani A, Bagheri Rekhne Z, Zareii S, Zarifian T (2025). Virtual/augmented reality for joint attention skills improvement in autism spectrum disorder: a systematic review. Int J Dev Disabil.

[23] Hocking DR, Farhat H, Gavrila R, Caeyenberghs K, Shields N (2019). Do active video games improve motor function in people with developmental disabilities? A meta-analysis of randomized controlled trials. Arch Phys Med Rehabil.

[24] Warsinsky S, Schmidt-Kraepelin M, Rank S, Thiebes S, Sunyaev A (2021). Conceptual ambiguity surrounding gamification and serious games in health care: literature review and development of game-based intervention reporting guidelines (GAMING). J Med Internet Res.

[25] Malihi M, Nguyen J, Cardy RE, Eldon S, Petta C, Kushki A (2020). Short report: evaluating the safety and usability of head-mounted virtual reality compared to monitor-displayed video for children with autism spectrum disorder. Autism.

[26] Wiebe A, Kannen K, Selaskowski B (2022). Virtual reality in the diagnostic and therapy for mental disorders: a systematic review. Clin Psychol Rev.

[27] Mittal P, Bhadania M, Tondak N (2024). Effect of immersive virtual reality-based training on cognitive, social, and emotional skills in children and adolescents with autism spectrum disorder: a meta-analysis of randomized controlled trials. Res Dev Disabil.

[28] Yang X, Wu J, Ma Y (2025). Effectiveness of virtual reality technology interventions in improving the social skills of children and adolescents with autism: systematic review. J Med Internet Res.

[29] Wu J, Xu Z, Liu H (2023). Effects of commercial exergames and conventional exercises on improving executive functions in children and adolescents: meta-analysis of randomized controlled trials. JMIR Serious Games.

[30] Page MJ, McKenzie JE, Bossuyt PM (2021). The PRISMA 2020 statement: an updated guideline for reporting systematic reviews. BMJ.

[31] Sterne JAC, Savović J, Page MJ (2019). RoB 2: a revised tool for assessing risk of bias in randomised trials. BMJ.

[32] Sterne JA, Hernán MA, Reeves BC (2016). ROBINS-I: a tool for assessing risk of bias in non-randomised studies of interventions. BMJ.

[33] Knapp G, Hartung J (2003). Improved tests for a random effects meta-regression with a single covariate. Stat Med.

[34] Borenstein M (2023). How to understand and report heterogeneity in a meta-analysis: the difference between I-squared and prediction intervals. Integr Med Res.

[35] Nagashima K, Noma H, Furukawa TA (2019). Prediction intervals for random-effects meta-analysis: a confidence distribution approach. Stat Methods Med Res.

[36] Sterne JAC, Sutton AJ, Ioannidis JPA (2011). Recommendations for examining and interpreting funnel plot asymmetry in meta-analyses of randomised controlled trials. BMJ.

[37] Guyatt G, Oxman AD, Akl EA (2011). GRADE guidelines: 1. Introduction-GRADE evidence profiles and summary of findings tables. J Clin Epidemiol.

[38] Fusar-Poli P, Radua J (2018). Ten simple rules for conducting umbrella reviews. Evid Based Ment Health.

[39] Nekar DM, Kang H, Alao H, Yu J (2022). Feasibility of using multiplayer game-based dual-task training with augmented reality and personal health record on social skills and cognitive function in children with autism. Children (Basel).

[40] Lee HK, Jin J (2023). The effect of a virtual reality exergame on motor skills and physical activity levels of children with a developmental disability. Res Dev Disabil.

[41] Miranda JM, Browne RAV, da Silva WQA, Rodrigues Dos Santos JP, Campbell CSG, Ramos IA (2025). Effects of a session of exergames and traditional games on inhibitory control in children with autism spectrum disorder: randomized controlled crossover trial. JMIR Serious Games.

[42] Abdel Ghafar MA, Abdelraouf OR, Harraz EM (2025). Virtual reality rehabilitation helps to improve postural balance in children with autism spectrum disorder: a randomized control trial. Phys Occup Ther Pediatr.

[43] Wu X, Liang J, Dong Y (2025). Effects of VR-based serious games on gross motor skills in chinese children with autism spectrum disorder in special education: a pilot study. J Autism Dev Disord.

[44] Ma X, Song K (2025). Rehabilitation therapy for children with autism based on interactive VR-motion serious game intervention: a randomized-controlled trial. Front Public Health.

[45] Oliveira Neves A da S, Soares MM, Marçal MA, de Souza Aarão TL (2026). The impact of using non-immersive virtual reality exergames on the motor skills of children with autism spectrum disorder. Int J Hum -Comput Interact.

[46] Anchieta MV, Torro-Alves N, da Fonsêca ÉKG, de Lima Osório F (2025). Effects of social skills training on social responsiveness of people with autism spectrum disorder: a systematic review with meta-analysis. Eur Child Adolesc Psychiatry.

[47] Altın Y, Boşnak Ö, Turhan C (2026). Examining virtual reality interventions for social skills in children with autism spectrum disorder: a systematic review. J Autism Dev Disord.

[48] O’Sullivan M, Kearney G (2018). Transforming Our World Through Design, Diversity and Education.

[49] Bachman JE, Fuqua RW (1983). Management of inappropriate behaviors of trainable mentally impaired students using antecedent exercise. J Appl Behav Anal.

[50] Papathomas P, Goldschmidt K (2017). Utilizing virtual reality and immersion video technology as a focused learning tool for children with autism spectrum disorder. J Pediatr Nurs.

[51] Alhowikan AM, Elamin NE, Aldayel SS (2023). Children with autism spectrum disorder exhibit elevated physical activity and reduced sedentary behavior. Brain Sci.

[52] Bremer E, Lloyd M (2021). Baseline behaviour moderates movement skill intervention outcomes among young children with autism spectrum disorder. Autism.

[53] Budavari AC, Pas ET, Azad GF, Volk HE (2022). Sitting on the sidelines: disparities in social, recreational, and community participation among adolescents with autism spectrum disorder. J Autism Dev Disord.

[54] Ruggeri A, Dancel A, Johnson R, Sargent B (2020). The effect of motor and physical activity intervention on motor outcomes of children with autism spectrum disorder: a systematic review. Autism.

[55] Wang H, Cheng G, Li MM (2025). The effectiveness and sustained effects of exercise therapy to improve executive function in children and adolescents with autism: a systematic review and meta-analysis. Eur J Pediatr.

[56] Jia Y, Zhou X, Tang Y, Li Q, Wang J, Fu Q (2025). Development of a virtual-reality eye movements-based system to assess basketball players’ decision making. Percept Mot Skills.

[57] Zhang Y, Li H, Xiao H (2025). The study of the effect of virtual reality technology combined with sports games on improving cognitive function in patients with brain injury: a meta analysis of randomized controlled trials. Front Neurol.

[58] Chen J, Zhou X, Wu X, Gao Z, Ye S (2023). Effects of exergaming on executive functions of children: a systematic review and meta-analysis from 2010 to 2023. Arch Public Health.

[59] Jia Y, Zhou X, Yang J, Fu Q (2024). Animated VR and 360-degree VR to assess and train team sports decision-making: a scoping review. Front Psychol.

[60] Caro K, Tentori M, Martinez-Garcia AI, Alvelais M (2017). Using the FroggyBobby exergame to support eye-body coordination development of children with severe autism. Int J Hum Comput Stud.

[61] Stanmore E, Stubbs B, Vancampfort D, de Bruin ED, Firth J (2017). The effect of active video games on cognitive functioning in clinical and non-clinical populations: a meta-analysis of randomized controlled trials. Neurosci Biobehav Rev.

[62] Feldman JI, Dunham K, Cassidy M, Wallace MT, Liu Y, Woynaroski TG (2018). Audiovisual multisensory integration in individuals with autism spectrum disorder: a systematic review and meta-analysis. Neurosci Biobehav Rev.

[63] Lecuyer A (2017). Playing with senses in VR: alternate perceptions combining vision and touch. IEEE Comput Graph Appl.

