# Supplementary material for: Effects of Virtual Reality–Based Physical Exercise Interventions on Behavioral, Executive Function, and Motor Outcomes in Children and Adolescents With Autism Spectrum Disorder: Systematic Review and Meta-Analysis
Source: J Med Internet Res. 2026 Jul 23;28:e98579. doi: 10.2196/98579 (PMC13394858; doi:10.2196/98579)
Supplement: Multimedia Appendix 1 [file jmir-v28-e98579-s001.docx]

**Supplementary Material**

**S1. Search strategy……………………………………2**

**S2. GRADE summary of findings table……. ……….5**

**S1. Search strategy**

| **Databases** | **Search strategy** | **N** |
| --- | --- | --- |
| **Pubmed**  **2026.05.07** | **(("Autism Spectrum Disorder"[Mesh] OR "Autistic Disorder"[Mesh] OR autism spectrum disorder*[tiab] OR ASD[tiab] OR autism[tiab] OR autistic[tiab] OR autistic disorder*[tiab] OR Asperger syndrome[tiab] OR pervasive developmental disorder*[tiab] OR PDD-NOS[tiab]) AND ("Virtual Reality"[Mesh] OR "Video Games"[Mesh] OR virtual reality[tiab] OR VR[tiab] OR augmented reality[tiab] OR AR[tiab] OR mixed reality[tiab] OR immersive technolog*[tiab] OR head mounted display*[tiab] OR HMD[tiab] OR exergame*[tiab] OR active video game*[tiab] OR motion-based game*[tiab] OR motion sensing game*[tiab]OR Kinect[tiab] OR Xbox Kinect[tiab] OR Nintendo Wii[tiab] OR Wii Fit[tiab] OR serious game*[tiab] OR virtual environment*[tiab] OR CAVE[tiab]) AND ("Exercise"[Mesh] OR "Motor Activity"[Mesh] OR "Sports"[Mesh] OR exercise*[tiab] OR physical activity*[tiab] OR physical exercise*[tiab] OR motor training[tiab] OR exercise intervention*[tiab] OR sport*[tiab] OR fitness training[tiab] OR balance training[tiab] OR aerobic exercise[tiab] OR cycling[tiab] OR dancing[tiab] OR movement training[tiab] OR motor skill training[tiab]))** | 67 |
| **Web of Science**  **2026.05.07** | ("autism spectrum disorder*"OR ASD OR autism OR autistic OR "autistic disorder*" OR "Asperger syndrome" OR "pervasive developmental disorder*" OR PDD-NOS) AND("virtual reality" OR VR OR "augmented reality" OR AR OR "mixed reality" OR exergame* OR "active video gam*" OR "motion-based gam*" OR "motion sensing gam*" OR Kinect OR "Xbox Kinect" OR "Nintendo Wii" OR "Wii Fit" OR "serious gam*" OR "virtual environment*" OR CAVE OR "head mounted display*" OR HMD)AND(exercise* OR "physical activity*" OR "physical exercise*" OR sport* OR "motor training" OR "exercise intervention*" OR "fitness training" OR "balance training" OR cycling OR dancing OR "movement training" OR "motor skill training") | 308 |
| **Scopus**  **2026.05.07** | ("autism spectrum disorder*" OR ASD OR autism OR autistic OR "autistic disorder*" OR "Asperger syndrome" OR "pervasive developmental disorder*" OR "PDD-NOS") AND("virtual reality" OR VR OR "augmented reality" OR AR OR "mixed reality" OR exergame* OR "active video gam*" OR "motion-based gam*" OR Kinect OR "Xbox Kinect" OR "Nintendo Wii" OR "Wii Fit" OR "serious gam*" OR "virtual environment*" OR CAVE OR HMD) AND (exercise* OR "physical activity*" OR "physical exercise*" OR sport* OR "motor training" OR "exercise intervention*" OR cycling OR dancing OR "balance training" OR "movement training") | 5652 |
| **PsycINFO**  **2026.05.07** | (DE "Autism Spectrum Disorders" OR autism spectrum disorder* OR ASD OR autism OR autistic) AND (DE "Virtual Reality" OR virtual reality OR augmented reality OR exergam* OR active video gam* OR serious gam* OR Kinect OR Wii) AND (DE "Exercise" OR DE "Physical Activity" OR exercise* OR physical activit* OR sport* OR motor training OR balance training) | 299 |
| **IEEE Xplore**  **2026.05.07** | (("All Metadata":"autism spectrum disorder" OR "All Metadata":ASD OR "All Metadata":autism)) AND ("All Metadata":"virtual reality" OR "All Metadata":"augmented reality" OR "All Metadata":exergame OR "All Metadata":"active video game" OR "All Metadata":"serious game" OR "All Metadata":Kinect OR "All Metadata":"Nintendo Wii") AND ("All Metadata":exercise OR "All Metadata":"physical activity" OR "All Metadata":"motor training" OR "All Metadata":"physical exercise") | 24 |
| **ProQuest**  **2026.05.07** | ("autism spectrum disorder*" OR ASD OR autism OR autistic) AND ("virtual reality" OR "augmented reality" OR exergame* OR "active video gam*" OR Kinect OR Wii OR "serious gam*") AND (exercise* OR "physical activity*" OR sport* OR "motor training") | 4587 |
| **S****cienceDirect**  **2026.05.07** | ("autism spectrum disorder" nOR ASD OR autism) AND ("virtual reality" OR "augmented reality" OR exergame OR "active video game" OR Kinect OR Wii) AND (exercise OR "physical activity" OR "motor training" OR sport) | 625 |
| **Embase**  **2026.05.07** | ('exp autism spectrum disorder'/exp OR autism spectrum disorder*:ti,ab OR ASD:ti,ab OR autism:ti,ab OR autistic:ti,ab) AND ('exp virtual reality'/exp OR virtual reality:ti,ab OR augmented reality:ti,ab OR exergame*:ti,ab OR active video game*:ti,ab OR serious game*:ti,ab OR Kinect:ti,ab OR Wii:ti,ab) AND ('exp exercise'/exp OR 'physical activity'/exp OR exercise*:ti,ab OR physical activity*:ti,ab OR sport*:ti,ab OR motor training:ti,ab)  (autism spectrum disorder OR autism spectrum disorder* OR ASD OR autism OR autistic) AND (virtual reality OR virtual reality OR augmented reality OR exergame* OR active video game OR serious game OR Kinect OR Wii) AND (exercise OR physical activity OR exercise OR physical activity OR sport* OR motor training) | 14 |
| **Total** | 1168 | 11576 |

S2. GRADE summary of findings table

| **Certainty assessment** | | | | | | | **№ of patients** | | **Effect** | | **Certainty** | **Importance** |
| --- | --- | --- | --- | --- | --- | --- | --- | --- | --- | --- | --- | --- |
| **№ of studies** | **Study design** | **Risk of bias** | **Inconsistency** | **Indirectness** | **Imprecision** | **Other considerations** | **Virtual Reality–Based Physical Exercise** | **usual care, no intervention, sham interventions** | **Relative (95% CI)** | **Absolute (95% CI)** |  |  |
| **Executive Function** | | | | | | | | | | | | |
| 4 | randomised trials | not serious | not serious | not serious | serious^a^ | none | 52 | 55 | - | SMD **0.75 SD higher** (0.32 higher to 1.18 higher) | ⨁⨁⨁◯ Moderate^a^ | CRITICAL |
| **Motor Outcomes** | | | | | | | | | | | | |
| 6 | randomised trials | not serious | serious^b^ | not serious | serious^a^ | none | 88 | 93 | - | SMD **1.08 SD higher** (0.08 higher to 2.08 higher) | ⨁⨁◯◯ Low^a,b^ | CRITICAL |

**CI:** confidence interval; **SMD:** standardised mean difference; **Explanations**: a. Sample size too small. b. I² > 70%
